# Oxamate enhances the efficacy of CAR-T therapy against glioblastoma via suppressing ectonucleotidases and CCR8 lactylation

**DOI:** 10.1186/s13046-023-02815-w

**Published:** 2023-09-29

**Authors:** Ting Sun, Bin Liu, Yanyan Li, Jie Wu, Yufei Cao, Shuangyu Yang, Huiling Tan, Lize Cai, Shiqi Zhang, Xinyue Qi, Dingjia Yu, Wei Yang

**Affiliations:** 1 Neurosurgery and Brain and Nerve Research Laboratory, The First Affiliated Hospital of Soochow University, Suzhou, Jiangsu China; 2 Department of Neurosurgery, Qinghai Provincial People’s Hospital, Xining, Qinghai China; 3 State Key Laboratory of Radiation Medicine and Protection, School of Radiation Medicine and Protection and Collaborative Innovation Center of Radiation Medicine of Jiangsu Higher Education Institutions, Soochow University, Suzhou, Jiangsu China

**Keywords:** CAR-T, Glioblastoma, Histone lactylation, Ectonucleotidases, CCR8

## Abstract

**Background:**

Chimeric antigen receptor (CAR)-T immunotherapy fails to treat solid tumors due in part to immunosuppressive microenvironment. Excess lactate produced by tumor glycolysis increases CAR-T immunosuppression. The mechanism of lactate inducing the formation of immunosuppressive microenvironment remains to be further explored.

**Methods:**

Immunocyte subpopulations and molecular characteristics were analyzed in the orthotopic xenografts of nude mice using flow cytometry assay and immunohistochemical staining after oxamate, a lactate dehydrogenase A (LDHA) inhibitor, and control T or CAR-T cells injection alone or in combination. RT-qPCR, western blot, flow cytometry, immunofluorescence, luciferase reporter assay, chromatin immunoprecipitation and ELISA were performed to measure the effect of lactate on the regulation of CD39, CD73 and CCR8 in cultured glioma stem cells, CD4 + T cells or macrophages.

**Results:**

Oxamate promoted immune activation of tumor-infiltrating CAR-T cells through altering the phenotypes of immune molecules and increasing regulatory T (Treg) cells infiltration in a glioblastoma mouse model. Lactate accumulation within cells upregulated CD39, CD73 and CCR8 expressions in both lactate-treated cells and glioma stem cells-co-cultured CD4 + T cells and macrophages, and intracellular lactate directly elevated the activities of these gene promotors through histone H3K18 lactylation.

**Conclusions:**

Utilizing lactate generation inhibitor not only reprogramed glucose metabolism of cancer stem cells, but also alleviated immunosuppression of tumor microenvironment and reduced tumor-infiltrating CAR-Treg cells, which may be a potential strategy to enhance CAR-T function in glioblastoma therapy.

**Supplementary Information:**

The online version contains supplementary material available at 10.1186/s13046-023-02815-w.

## Background

Glioblastoma multiforme (GBM) is the most common and lethal primary brain tumor of the central nervous system. The standard treatment of GBM consists of surgical resection, radiotherapy and chemotherapy with temozolomide, while the median survival rate is less than 15 months upon diagnosis [1]. The negative prognosis and subsequent recurrence of GBM are partially attributed to the presence of glioma stem cells (GSCs), a small subpopulation of GBM with self-renewal ability and therapeutic resistance [2]. The complex interplay from different components in GBM forms an immunosuppressive tumor microenvironment (TME). Although a certain amount of immune cells infiltrates into tumor, there are high ratios of regulatory B and T cells as well as immunosuppressive myeloid cells [3]. GCSs contribute to immunosuppression within the TME, damaging GSCs is the key to overcome the immunotherapeutic resistances.

Chimeric antigen receptor-T (CAR-T) cells therapy provides a new personalized and immunotherapeutic approach on cancer treatment in recent years. The clinical successes of CD19 CAR-T cells against leukemia provide great encouragement for the further development of antigen specific CAR-T therapy [4]. Several clinical studies have demonstrated notable anti-tumor effects of CAR-T therapy on GBM [5, 6]. However, immunosuppressive TME may be one of the main obstacles for CAR-T cells to exert their functions [7]. Single cell mRNA-seq data showed a higher frequency of CD4 + CAR-T cells expressing regulatory T (Treg) cell phenotype, which was associated with disease progression. Combination of CAR-Treg and lactate dehydrogenase (LDH) levels was superior for prediction of clinical response on cancer patients than each feature alone [8].

Cancer cells metabolize most glucose carbon to lactate via anaerobic glycolysis instead of mitochondrial oxidative phosphorylation for energy generation to enhance self-proliferation and survival in the presence of oxygen, known as the Warburg effect [9]. Excess lactate production offers growth benefits to cancer cells for aggressiveness, migration, angiogenesis, immunosuppression and resistance to therapy [9]. Cancer growth activates key genes of glycolytic phenotype in response to TME changes, and upregulated LDH leads to the increase of lactate production [10]. LDH is constituted by LDHA and LDHB subunits to form tetramer and found as five different isoforms (LDH1-5). LDHA is closely associated with various human metabolic cancer, and LDHA inhibitors discovered so far include substrate (pyruvate) competitive inhibitors, cofactor (NADH) competitive inhibitors and dual competitive inhibitors [10]. Oxamate, an analog of pyruvate or isosteric of pyruvate [11], was used to inhibit LDHA activity by competition with pyruvate in this study. Blockade of the Warburg effect to reprogram lactate metabolism in the TME has been proposed to be a promising therapy for treating cancer [11].

Epigenetics focuses on the modulation of gene expression without changing the sequence of genomic DNA, usually including modifications of histone and DNA through acetylation, lactylation, and methylation. Epigenetic modifiers play critical roles in regulating intrinsic immunogenicity of cancer cell and T cell function [12]. Recently, several findings have revealed that histone lactylation, addition of lactyl groups to the lysine residues of histones [13], regulated gene transcription in cancer [14] and immune cells [13, 15]. Histone lactylation formed by intracellular lactate at Histone H3 lysine K18 lactylation (H3K18la) sites directly promotes gene transcription, and lactate level may influence the extent of histone lactylation [13]. Despite confirming the roles of histone lactylation in the alteration of cellular function, the molecular events in lactylation-induced immunosuppression have yet to be elucidated.

In the current study, we investigated the efficacy of lactate dehydrogenase A (LDHA) inhibition and CAR-T cells in combination against GBM. The decrease of lactate induced by oxamate changed immune associated molecule expressions and immunocytes infiltration in TME. We explored the function of lactate as an epigenetic regulation factor on ectonucleotidases CD39 and CD73, switching ATP to adenosine, and chemokine (C-C motif) receptor 8 (CCR8), a marker of tumor-infiltrating Treg cells, demonstrating the potential of inhibiting lactate production as a strategy for improving CAR-T cells function in GBM treatment.

## Methods

### Cell culture

GSCs 51 A, 66 A, SHG141A and SHG142A isolated from GBM patients and identified using CD133 and Nestin antibodies as previously described [16] were cultured in DMEM/F12 medium supplemented with 2% B27, 20 ng/mL epidermal growth factor and basic fibroblast growth factor. Human monocytic THP-1 cell line was purchased from American Type Culture Collection (ATCC). THP-1 cells were cultured in RPMI 1640 medium supplemented with 10% FBS, and differentiated into macrophages by 50 ng/ml phorbol-myristate acetate (PMA) treatment for 24 h (Supplementary Fig S1A). All cells were cultured in a cell incubator at 37 °C with 5% CO2, and have been tested for mycoplasma contamination.

### T cells isolation

Human whole blood was obtained from three healthy donors with informed consent, which was approved by the Ethics Committee of First Affiliated Hospital of Soochow University (2020420). Peripheral blood mononuclear cells (PBMCs) were isolated from blood by Ficoll-Hypaque gradient centrifugation. EasySep™ Human T Cell Isolation Kit were used to purify T cells from PBMCs for control T and CAR-T cell preparation. EasySep™ Human CD4 + T Cell Isolation Kit was used to obtain CD4 + T cells. T lymphocytes were cultured in RPMI 1640 containing 10% FBS with 300 U/ml IL-2 for 7 days, and 300 U/ml IL-2 was added every two days. Cells were activated by 5 µg/ml anti-human CD28 antibody and 1 µg/ml anti-human CD3 antibody. The purities of isolated cells were more than 85% CD3 + and CD4 + population by flow cytometry, respectively (Supplementary Fig S1B and S1C).

### CAR-T cell preparation

CAR vector design and CAR-T cell preparation were as previously described [17]. Briefly, the CAR targeting domain directs to EGFRvIII antibodies single-chain variable fragments (scFvs) derived from human 139 antibody includes a CD8 hinge spacer, transmembrane domain 4-1BB and CD3ζ endo-domains.

CD3 + T cells were transfected with lentivirus carrying CAR structure, then the EGFRvIII CAR-T cells were maintained at the density of 10^6^ cells/ml and used for anti-tumor immune response experiments 10 days after CAR transfection.

### Animal model

All animal experiments were conducted in accordance with protocols and approved by the Institutional Animal Care and Use Committee of Soochow university. Male BALB/c nude mice of 6-8-week-old were housed under controlled temperature and humidity with a 12 h light-dark cycle. Mice was randomly grouped by body weight in the experiments.

**Fig. 1:**
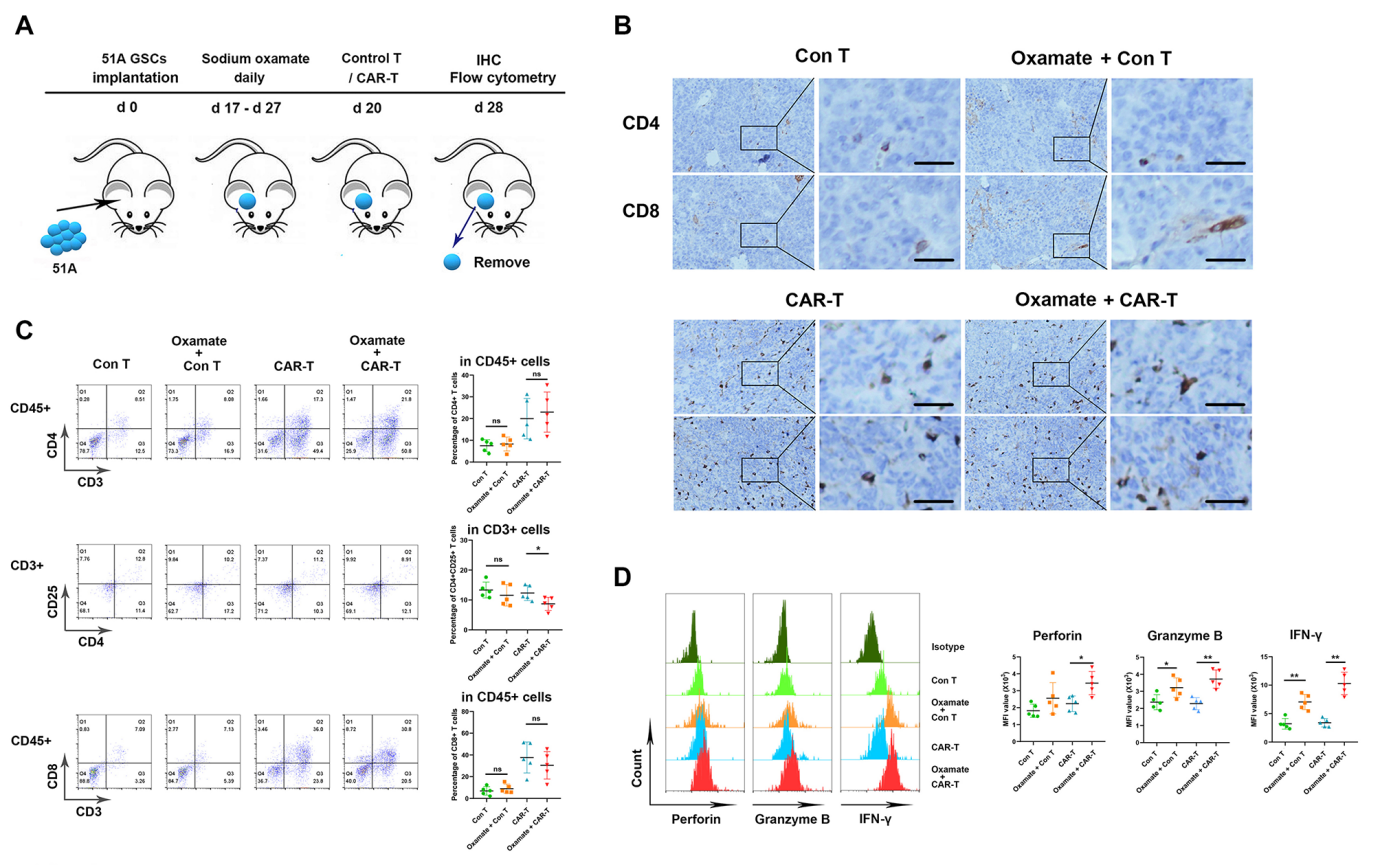
Oxamate enhanced the function of TILs in both control T and CAR-T cells inoculation. 51 A GSCs were orthotopically inoculated into nude mice to establish glioma model. The tumor bearing mice were injected with sodium oxamate with or without control T or CAR-T cells. Tumors were removed from the mice at day 28, then prepared into sections for IHC staining or single cell suspensions for flow cytometry assay. (**A**) Timeline of tumor-bearing mouse models with treatment schedule. (**B**) Representative images of formalin-fixed paraffin embedded sections from xenografts stained using anti-CD4 and CD8 antibodies (×200). Scale bar represents 50 μm. (**C**) All lymphocytes were gated as CD45 + CD3 + cells. The percentages of CD4 + and CD8 + T cells within the CD45 + cells and CD4 + CD25 + T cells within the CD4 + cells. (**D**) The median fluorescence intensity (MFI) of perforin, granzyme B, and IFN-γ in CD8 + T cells. N = 5, *P < 0.05, **P < 0.01

For the orthotopic mouse model, 10^5^ of 51 A GSCs in 5 µl PBS were injected into the frontal lobe of the mouse cerebrum via stereotactic injection. The coordinates of GSCs inoculation were 2 mm to the right of the midline and 1 mm anterior to the bregma at a depth of 3 mm. The mice were randomly divided into 4 groups. (1) Control T group, mice were injected with 10^7^ control T cells in 100 ul via tail vein 20 days after 51 A cells implantation; (2) Oxamate plus control T group, mice were intraperitoneally inoculated with 300 mg/kg sodium oxamate [18] 17 days after 51 A cells implantation for 3 weeks and injected with control T cells; (3) CAR-T group, mice were injected with 10^7^ EGFRvIII CAR-T cells in 100 ul via tail vein 20 days after 51 A cells implantation; (4) Oxamate plus CAR-T group, mice were administrated with sodium oxamate and CAR-T cells in combination (Fig. 1A). The tumor-bearing mice were sacrificed with carbon dioxide 28 days after GSCs inoculation, and tumor tissue of each mouse was collected for the analysis of immunocyte subpopulations and molecular characteristics using flow cytometry assay and brain section staining.

For CD73 activation, GSCs 51 A were transfected with lentivirus carrying vector or CD73 cDNA, then inoculated into the brain of nude mice to establish orthotopic xenografts. The mice were inoculated with sodium oxamate and CAR-T cells in combination. When severe neurological symptoms appeared, the tumor-bearing mice were sacrificed and the survival was recorded. Tumor tissues were collected for section staining.

**Fig. 2:**
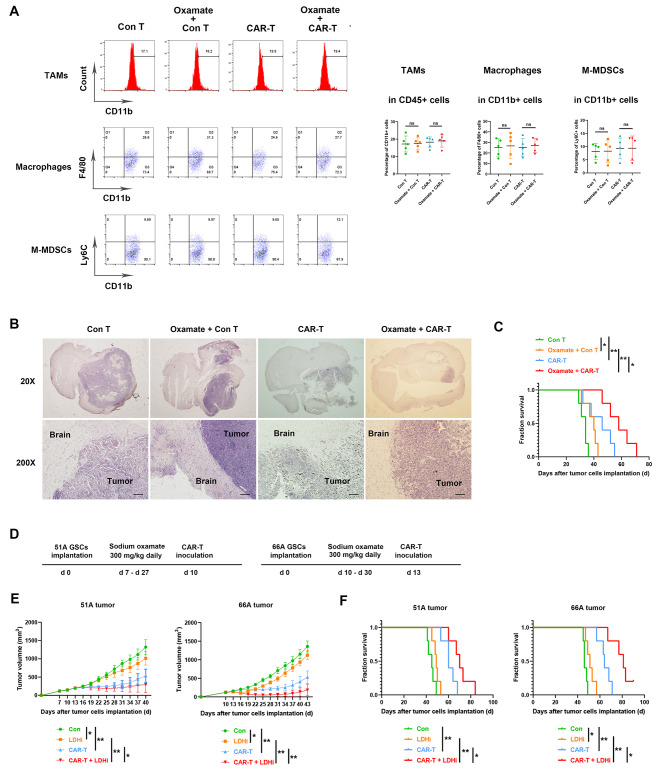
Oxamate unchanged myeloid subpopulation in tumor tissue while reduced tumor growth. (**A**-**C**) 51 A GSCs were orthotopically inoculated into nude mice and tumors were removed from the mice at day 28. (**A**) Tumors were dissociated into single cells for flow cytometry assay. The classification of myeloid subpopulations was gated in CD45 + CD11b + cells. The percentages of TAMs (CD11b+), macrophages (CD11b + F4/80+), and M-MDSCs (CD11b + Ly6C+) within CD45 + cells in tumor tissues were analyzed. (**B**) Paraffin sections from the whole brain were stained with H&E and representative images showed tumor location and size. Scale bar represents 100 μm. (**C**) Survival of tumor-bearing nude mice. (**D**-**F**) Nude mice with subcutaneous tumor were injected with saline, sodium oxamate, CAR-T or sodium oxamate plus CAR-T combination. (**D**) Timeline of tumor-bearing mice with treatment schedules. (**E**) Subcutaneous tumor size in different time points after GSCs implantation. (**F**) Kaplan–Meier curves showing mice survival. N = 5, *P < 0.05, **P < 0.01

For the subcutaneous mouse model, 1 × 10^6^ GSCs 51 or 66 A in 100 µl were injected into right axilla. When the tumor diameter reached 5 mm, the mice were randomly divided into 4 groups. (1) Control group, mice were intraperitoneally administrated with saline as a control; (2) Oxamate group, mice were intraperitoneally injected with 300 mg/kg sodium oxamate from the day of grouping for 3 weeks; (3) CAR-T group, mice were injected with 10^7^ EGFRvIII CAR-T cells in 100 ul via tail vein 3 days after grouping; (4) Oxamate plus CAR-T group, mice were administrated with sodium oxamate and CAR-T cells in combination (Fig. 2E). Tumor diameters were measured every 3 days with a caliper, and tumor volumes were calculated using the following formula: width^2^ × length/2 = V (mm^3^).

### Preparation of single cell suspension and marker staining

Tumor tissues from nude mice were collected and mechanically dissociated into single cell suspensions using the Tumor Dissociation Kit on a gentleMACS™ Dissociator (Miltenyi Biotec) according to manufacturer’s instructions. The suspension passed a 70 μm mesh cell strainer to move undigested cell and tissue masses. Red blood cells were lysed in ACK lysing buffer. Cells were resuspended in Percoll gradients and centrifuged at 2,000 rpm for 20 min to separate mononuclear cells. 10^6^ cells in 1 ml were used for marker analysis. PMA, ionophore and protein transport inhibitor were added into cell suspension for 5 h incubation. For lymphocyte subpopulations and immunosuppressive markers analyses, cells were stained with anti-CD45 BV605, anti-CD3 BV711, anti-CD4 PerCP Cy5.5, anti-CD8 BV510, anti-CD25 eFluor 450, anti-CD73 FITC, anti-CD39 PE and anti-CCR8 BV786. For cytokines analyses, cells were stained with anti-CD45 BV605 and anti-CD8 BV510, then fixed and permeabilized using BD Cytofix/Cytoperm™ Plus Fixation/Permeabilization Kit for anti-IFN-γ BV650, anti-perforin APC, and anti-granzyme B PE. For myeloid cells analyses, cells were stained with anti-CD11b FITC, anti-CD45 BV605, anti-F4/80 BV650, anti-Ly6C APC, anti-human CD73 PE, anti-mouse CD39 PE-Cy7, anti-mouse CCR8 BV711, and anti-human CD39 PerCP-eFluor 710. Then cells were stained using LIVE/DEAD Fixable Blue Cell Stain Kit. Anti-mouse IgG was used as a negative control. 5 × 10^3^ cells were collected for cell subpopulations and phenotypes analyses using flow cytometry.

### Immunohistochemistry (IHC) and immunofluorescence (IF) staining

Brain tissue from mice were dissected, then fixed in 10% formalin, dehydrated in ethyl alcohol, cleaned in xylene, embedded in paraffin and sectioned at a thickness of 4 μm. The slides were performed by H&E staining or incubated with primary antibodies including anti-CD4 or anti-CD8 overnight at 4 °C, and then incubated with a horseradish peroxidase-conjugated secondary antibody at room temperature. The slices were stained with a diaminobenzidine substrate, and images in every sample were captured under optical microscope.

For IF staining, primary anti-CD73 antibody was incubated on slides overnight at 4 °C, then secondary antibody labeled by Alexa Fluor 555 was used. Cells were counterstained with DAPI and analyzed by fluorescence microscopy.

### Flow cytometry

Cultured cells in vitro were stained with PE-conjugated mouse anti-CD39, CD73, CCR8, CTLA-4 and Tim-3 monoclonal antibodies in the dark at 4 °C for 1 h, then analyzed using a flow cytometer (BD Bioscience). PE-conjugated mouse IgG was used as a control.

Cells were stained using PE Annexin V apoptosis detection kit with 7-AAD according to manufacturer’s instructions, then dead cells were detected using a flow cytometer.

### Co-culture of GSCs and CD4 + T or THP-1 cells

Cell culture inserts were used for co-culture of GSCs and CD4 + T or THP-1 cells. CD4 + T or THP-1 cells in 1 × 10^5^ cells/200 µl were inoculated into the upper chambers with RPMI 1640 (serum-free). GSCs in 5 × 10^4^ cells/500 µl were inoculated into the lower chambers with stem cell complete medium. Cells were co-cultured for 12 h, then CD4 + T or THP-1 cells in the upper chambers were collected for the measurement of CD39 and CCR8, CCL1 and CCL18 expressions.

### CAR-T cytotoxicity

GSCs were stained with CFSE in advance, then 10^5^ GSCs were co-cultured with CAR-T at an effector : target (E:T) ratio of 5:1 in stem cell complete medium for 16 h in the existence of PBS or sodium oxamate. Cells were collected and stained by 7-AAD for 15 min at room temperature, then analyzed using flow cytometry. The cytotoxicity of CAR-T was shown by the percentage of double positive cells in CFSE positive cells.

### Lactate measurement

GSCs were seeded into 96-well plates at a density of 2 × 10^4^ cells/well in 100 ul and treated with or without 20 mM sodium oxamate for 24 h. Supernatants were collected for lactate detection. To measure intracellular lactate production, cells were centrifuged and homogenized in lactate assay buffer. Lactate level was measured by using lactate colorimetric assay kit according to manufacturer’s protocol. Absorbance was measured in a microplate reader at OD 570 nm, and intracellular lactate were normalized by the cell numbers.

### Enzyme-linked immunosorbent assay (ELISA)

Supernatants from cultured cells were collected and the concentrations of IFN-γ, granzyme B, IL-6 and IL-17 A were measured using ELISA kits following the manufacturer’s instructions.

### Luciferase reporter assay

Macrophages and CD4 + T cells were transfected with pGL3-CD39 promotor plasmid, GSCs were transfected with pGL3-CD73 promotor plasmid and CD4 + T cells were transfected with pGL3-CCR8 promotor plasmid, then treated with sodium lactate or sodium oxamate. Each gene promotor of 2000 bp ahead of transcription initiation site was inserted pGL3 plasmid. Relative luciferase units were measured using the Dual-Glo Luciferase Assay System according to the manufacturer’s instructions. Relative luciferase units from firefly luciferase signal were normalized to Renilla signal.

### Western blot

Total protein was extracted using cell lysate at 4 °C and quantified using a BCA Protein Assay Kit. Then equal amounts of proteins were separated on 12% sodium dodecyl sulfate-polyacrylamide gel electrophoresis (SDS-PAGE) and transferred to a polyvinylidene fluoride (PVDF) membrane. After blocking with 5% fat-free milk for 1 h, the membrane was incubated with primary antibodies at 4 °C overnight and secondary antibodies for 2 h at room temperature. The protein was analyzed by an enhanced chemiluminescence kit and exposed in darkness.

### Chromatin immunoprecipitation (ChIP)

ChIP assay was performed using Chromatin IP Kit according to manufacturer’s protocol. Briefly, cells were fixed with 37% formaldehyde and lysed, then DNA was sonicated to appropriate fragments. Chromatin fragment of 10 µg was mixed with H3K18la antibody at 4 ℃ overnight. Protein G magnetic beads of 30 µl were added and incubated for 2 h at 4 ℃. Chromatin was eluted from beads, and the specific DNA fragments were determined using qPCR reactions. The primer sequences are shown in Supplementary Table S1.

### Reverse transcription and quantitative real-time reverse transcription-polymerase chain reaction (RT-qPCR)

Total RNA was extracted from GSCs, macrophages or CD4 + T cells using TRIzol reagent. The first strand of cDNA was synthesized using the total RNA, then qPCR amplification was carried out using the obtained cDNA template. The expression levels of detected genes were calculated by the 2-ΔΔCt method. GAPDH was the internal control, and the primer sequences of detected genes were listed in Supplementary Table S1.

### Statistical analysis

All the experiments in vitro were repeated at least three times. The statistical analysis was performed by a one-way ANOVA or unpaired two-tailed Student’s t test using GraphPad prism 8 software. Survival time of animal was analyzed by Kaplan–Meier process. Error bars represent the SD values and statistical significance is considered as *P < 0.05, **P < 0.01.

## Results

### Oxamate changed the phenotypes of infiltrated CAR-T

To determine whether oxamate alters the immune function of tumor-infiltrating EGFRvIII CAR-T cells in intracranial tumors, we orthotopically inoculated 51 A GSCs positively expressing EGFRvIII [17] into nude mice and analyzed the composition and phenotype of immune cells in tumor tissues. Immunohistological results showed no changes in the numbers of CD4 + and CD8 + tumor-infiltrating lymphocytes (TILs) between oxamte plus CAR-T combination and CAR-T alone groups (Fig. 1B). Flow cytometry confirmed that the percentages of CD4 + T and CD8 + T and in infiltrating CD3 + cells of each CAR-T-administrated tumor were no differences, however, the percentages of Treg cells were decreased by oxamate administration (Fig. 1C). Furthermore, a significant increase in cytotoxic and IFN-γ + CD8 + T cells in resected tumors was shown in oxamate plus CAR-T combination comparing to CAR-T alone group (Fig. 1D). These data suggested that oxamate enhanced the activation and cytotoxicity of CAR-T cells. Consistently, oxamate plus control T cells administration increased the activity of TILs comparing with control T cells alone (Fig. 1A-D).

### Oxamate unchanged the ratio of myeloid cells while inhibited tumor growth

To determine whether oxamate might impact the infiltrating myeloid cells within tumors, we analyzed the characteristics of myeloid cells after tumor-bearing mice were treated with oxamate. The percentages of tumor-associated macrophages (TAMs), macrophages and monocytic myeloid-derived immunosuppressive cells (M-MDSCs) were unchanged between either control T cells and oxamate plus control T cells groups or CAR-T and oxamate plus CAR-T groups (Fig. 2A). However, a smaller tumor size (Fig. 2B) and longer survival (Fig. 2C) were observed in oxamate plus CAR-T comparing with CAR-T alone group.

To address whether oxamate enhances the anti-tumor effect of immunotherapy in multiple tumor cell GBM models, we subcutaneously inoculated GSCs into nude mice to test the efficacy of oxamate and CAR-T. Both oxamate and CAR-T injection alone resulted in growth inhibition of both 51 and 66 A tumor. The inhibitory effect was further elevated by combination of oxamate and CAR-T (Fig. 2E). Furthermore, extended survival of tumor-bearing mice was showed in oxamate plus CAR-T comparing with CAR-T alone group (Fig. 2F). These data confirmed that LDHA inhibition by oxamate enhanced the anti-tumor effect of CAR-T cells.

### LDHA expression was negatively relative to glioma patient prognosis

To investigate the potential use of oxamate in glioma therapy, we analyzed the expressions of LDHs in GBM and low-grade glioma (LGG) in The Cancer Genome Atlas (TCGA) datasets. High expression of LDHA, a main LDH subunit, in GBM tissues was found (Supplementary Fig S2A), and higher level of specific LDHA was associated with a significantly poorer outcome in GBM and LGG patients (Supplementary Fig S2B).

To reveal LDHA expression profiles in human GBM, we analyzed the different cell populations (left panel) and corresponding LDHA transcriptomic expression (right panel) from the data of single-cell RNA-seq on the UCSC Cell Browser [19]. We observed higher expressions of LDHA in human GBM samples, especially in astrocytes, radical glia, progenitor, microglia, tumor associated microphage and endothelial cells (Supplementary Fig S2C), supporting LDHA as a potential target for GBM therapy.

### Lactate inhibited the pro-inflammatory function of T lymphocytes

Oxamate inhibits the production of lactate, and lactate released into the microenvironment may be one of the main reasons of CAR-T exhaustion. Given that CAR-T is generated by T cell modification, we directly examined the effect of lactate on in vitro cultured T lymphocytes. CD4 + and CD8 + T lymphocytes were treated with 10 mM lactic acid or sodium lactate for 24 h, then we found that both treatment increased CTLA-4 and PD-1 and decreased ICOS and Ki-67 expressions in cultured T cells (Supplementary Fig S3A). Meanwhile, reduced IFN-γ and unchanged IL-10 secretion was observed in supernatants (Supplementary Fig S3B). In addition, lactic acid caused a slightly increased death of T cells (Supplementary Fig S3C).

### Lactate induced histone lactylation elevating CD39 transcription

**Fig. 3:**
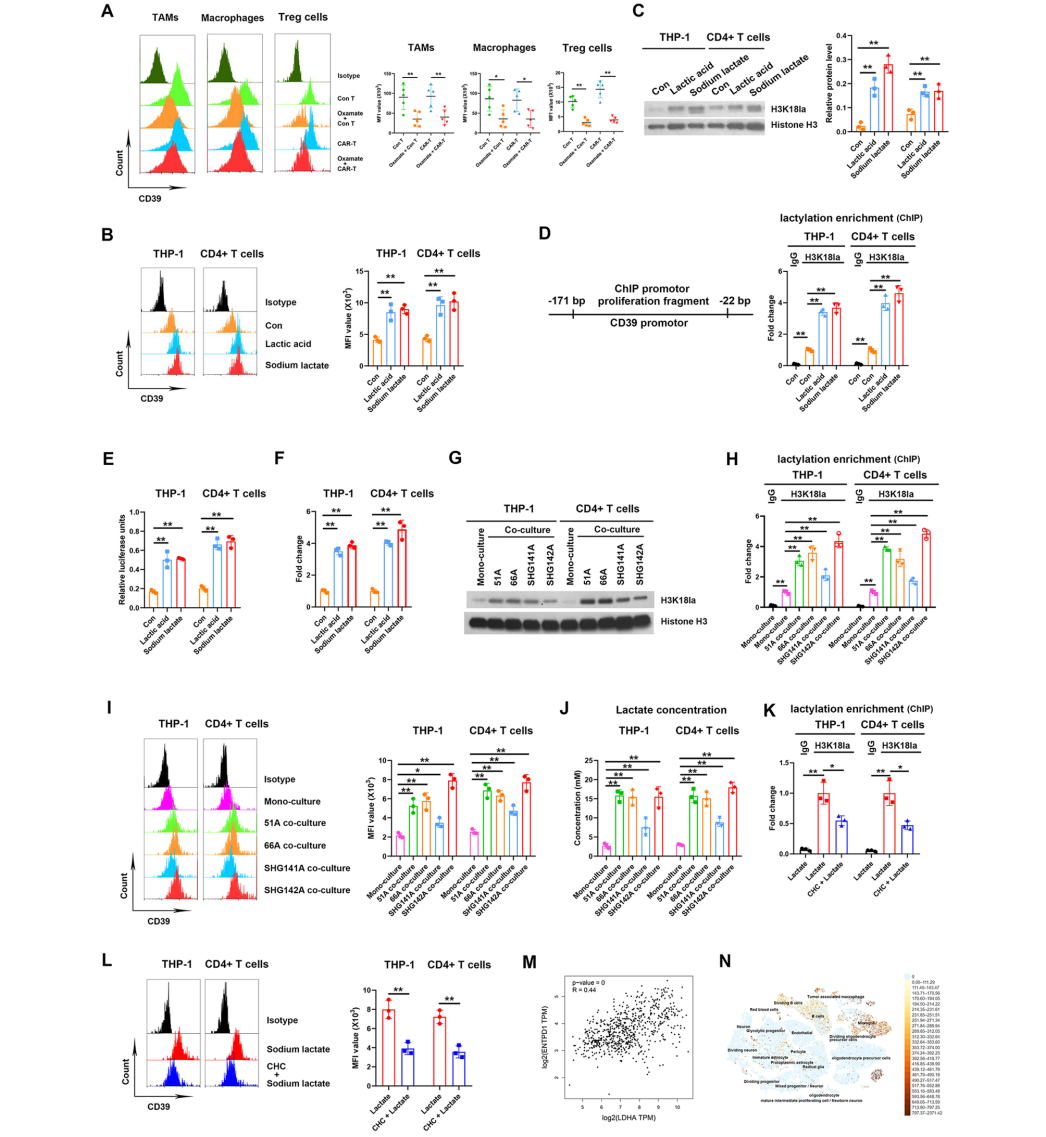
Lactate in the TME increased CD39 level by inducing H3K18la on gene promoter. (**A**) CD39 levels in TAMs, macrophages and Treg cells within tumor tissues from 51 A GSCs implanted nude mice. The gating strategies of TAMs and macrophages in Fig. 2A and Treg cells in Fig. 1C were showed. N = 5. (**B**-**F**) Macrophages from differentiated THP-1 cells and CD4 + T lymphocytes isolated from PBMCs were treated with PBS, 10 mM lactic acid or sodium lactate for 24 h. (**B**) CD39 protein level was detected using flow cytometry. (**C**) Histone H3K18la level was detected using western blot. (**D**) ChIP assay was performed using anti-H3K18la antibody, and the enrichment of CD39 gene promotor was qualified by qPCR. (**E**) Lymphocytes were transfected with pGL3 plasmid carrying CD39 promotor sequence, luciferase units were detected after lactic acid or sodium lactate treatment. (**F**) CD39 mRNA expression was detected using RT-qPCR. (**G**-**J**) THP-1 and CD4 + T cells were mono-cultured or co-cultured with GSCs for 12 h, H3K18la levels (**G**), H3K18la enrichment on CD39 promoter region (H) and CD39 protein level (**I**) in THP-1 and T cells and lactate concentration in medium (**J**) were detected. (**K**, **L**) THP-1 and CD4 + T cells were treated with sodium lactate for 24 h in pretreatment with 3 mM CHC for 2 h or not, H3K18la enrichment on CD39 promoter region using ChIP assay (**K**) and CD39 protein level (**L**) were detected. (**M**) The correlation of LDHA and ENTPD1, coding CD39 protein, expressions in TCGA database. (**N**) CD39 expression in GBM patients using single-cell RNA-seq on the UCSC Cell Browser. *P < 0.05, **P < 0.01

Ectonucleosidases CD39 and CD73 on cell surface play critical roles in forming an immunosuppressive TME [20]. We found that oxamate decreased the level of CD39 in TAMs, macrophages and Treg cells within 51 A GSCs grafts of nude mice both in control T and CAR-T groups (Fig. 3A). Consistently, both lactic acid and sodium lactate increased CD39 expression in THP-1 and CD4 + T cells in vitro (Fig. 3B). Lactate directly modifies nuclear histone proposed as histone lactylation, a process of post-translational modification in macrophages [13] and CD4 + T cells [21]. Consistent with previous results, we found that both lactic acid and sodium lactate increased the histone H3K18la levels in THP-1 and CD4 + T cells in vitro, and antibodies detecting total histone H3 served as a control (Fig. 3C). To verify that whether histone lactylation is a potential mechanism regulating CD39 expression, ChIP was performed using anti-H3K18la antibodies, and the data showed that H3K18la was enriched in the promoter regions of CD39 (Fig. 3D), suggesting direct regulation of lactyl-histone on CD39 promotor. Meanwhile, higher activity of CD39 gene promotor by luciferase reporter assay (Fig. 3E) and upregulated CD39 mRNA expression (Fig. 3F) were confirmed after lactic acid and sodium lactate treatment.

To investigate the effect of tumor-derived lactate on macrophages and T lymphocytes, GSCs were co-cultured with THP-1 and CD4 + T cells. The data showed higher H3K18la levels using western blot in co-cultured system comparing to T cell mono-culture alone (Fig. 3G). Furthermore, ChIP assay showed H3K18la enrichments in the promoter regions of CD39 (Fig. 3H). Meanwhile. the expressions of CD39 were upregulated (Fig. 3I), accompanying increased lactate concentrations in media (Fig. 3J). Moreover, oxamate decreased H3K18la level, H3K18la enrichments on CD39 promoter, CD39 expression and lactate concentration when 51 A GSCs were co-cultured with THP-1 or CD4 + T cells (Supplementary Fig S4). These data suggested that tumor-derived lactate upregulated CD39 expression via histone lactylation.

To explore whether macrophages and T cells uptake lactate through monocarboxylic acid transporters (MCTs) for CD39 lactylation, MCT inhibitor (CHC) was used to block extracellular lactate entrance. MCTs blockade suppressed lactate-induced H3K18la enrichment on CD39 promotor region (Fig. 3K) and CD39 expression (Fig. 3L), suggesting lactate in the TME promoted H3K18la of CD39 promotor through MCTs. Moreover, the analysis in TCGA database consistently demonstrated a positive correlation on LDHA and CD39 expressions in glioma patients (Fig. 3M). The data of single-cell RNA-seq on the UCSC Cell Browser also showed high expressions of CD39 in microglia and tumor associated microphage in human GBM samples [19] (Fig. 3N). Collectively, lactate was partly responsible for CD39 upregulation due to H3K18la enrichment in CD39 promotor region.

### Lactate directly promoted H3K18la enrichment on CD73 promotor

**Fig. 4:**
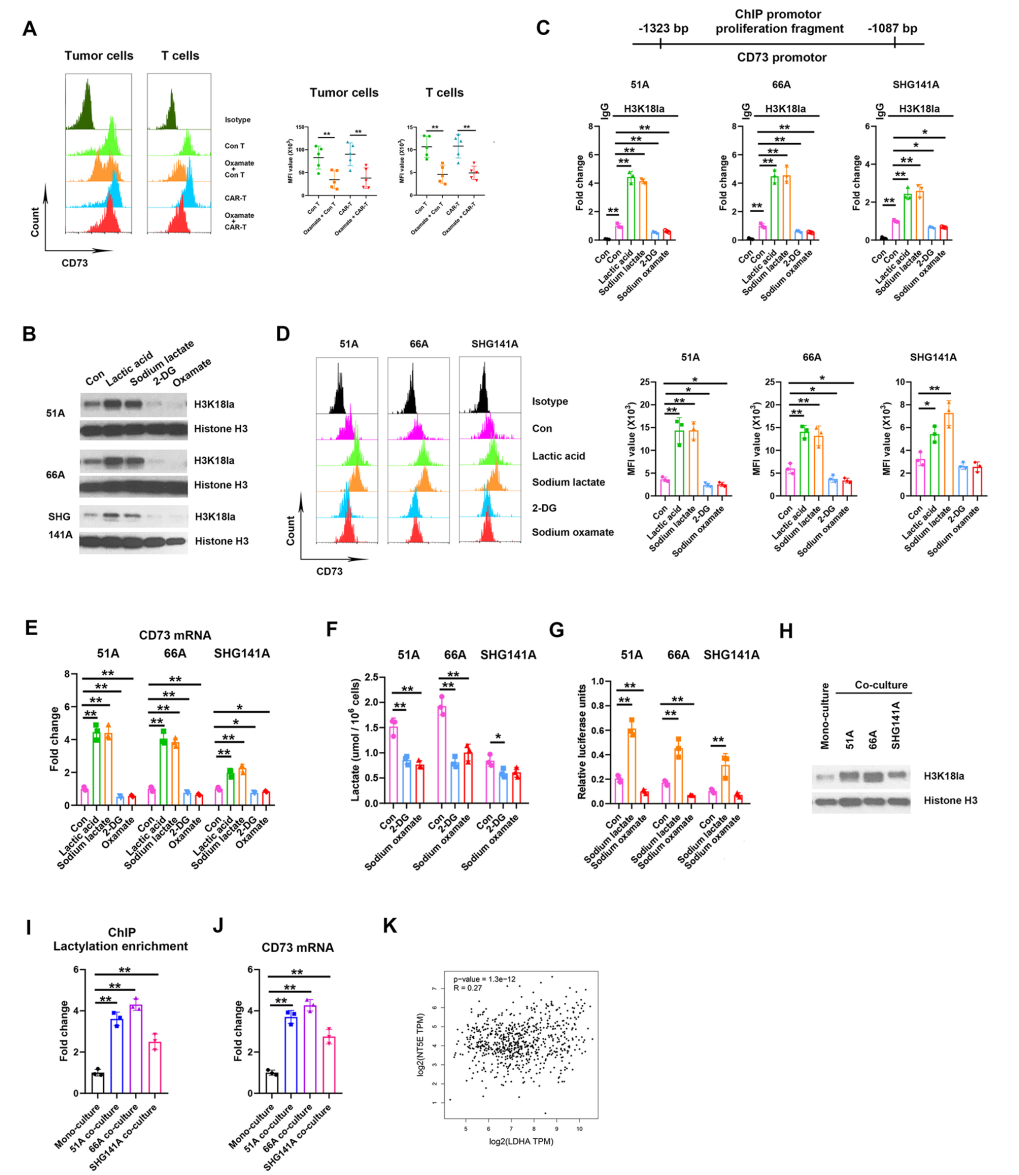
Lactate increased CD73 expression by H3K18la in GSCs. (**A**) CD73 levels in tumor and T cells within the xenografts of nude mice. N = 5. GSCs were treated with PBS, 10 mM lactic acid, 10 mM sodium lactate, 2 mM 2-DG or 20 mM sodium oxamate for 24 h, H3K18 lactylation using western blot (**B**), H3K18la enrichment on CD73 promotor using ChIP (**C**), CD73 protein level using flow cytometry (**D**), CD73 mRNA expression using RT-qPCR (**E**), and intracellular lactate production using colorimetric assay (**F**) were measured. (**G**) GSCs were transfected with pGL3-CD73 promotor plasmid for 24 h, then treated with PBS, sodium lactate or sodium oxamate, the luciferase activities were measured by dual-luciferase reporter system. T cells were mono-cultured or co-cultured with GSCs for 12 h, H3K18la levels (**H**), CD73 mRNA expression (**I**) and H3K18la enrichment on CD73 promoter region (**J**) in T cells were detected. (**K**) The correlation of LDHA and NT5E, editing CD73 protein, expressions in TCGA database. *P < 0.05, **P < 0.01

In intracranial grafts, CD73 expressions on the surface of tumor and T cells were declined by oxamate administration (Fig. 4A). We next investigated whether the intracellular lactate regulated CD73 expression through histone lactylation. Both lactic acid and sodium lactate treatment significantly increased the H3K18la levels (Fig. 4B) and H3K18la enrichment on CD73 promotor (Fig. 4C) in multiple GSCs comparing to control, and CD73 protein levels (Fig. 4D) and mRNA expressions (Fig. 4E) were consistently elevated. Furthermore, we used 2-deoxy-D-glucose (2-DG), a glucose analog that suppresses normal glucose metabolism, to inhibit endogenous lactate production. Both 2-DG and sodium oxamate treatment downregulated H3K18la levels (Fig. 4B), H3K18la enrichments on CD73 promotor (Fig. 4C) and CD73 expression (Fig. 4D), accompanying decrease of intracellular lactate level (Fig. 4F). In addition, luciferase reporter assay consistently confirmed that sodium lactate enhanced while sodium oxamate reduced CD73 promotor activity (Fig. 4G). We co-cultured GSCs and T cells and consistently confirmed higher H3K18la (Fig. 4H), CD73 mRNA level (Fig. 4I) and CD73 promotor activity (Fig. 4J) in co-cultured T cells comparing with mono-culture. Moreover, oxamate decreased H3K18la level, H3K18la enrichments on CD73 promoter and CD73 expression in co-cultured condition of 51 A GSCs and T cells (Supplementary Fig S5). Gene correlation analysis also showed that LDHA and CD73 were positively correlative in GBM patients (Fig. 4K). These data suggested that intracellular lactate increased CD73 expressions by H3K18la.

### CD73 upregulation decreased the antitumor effect of oxamate and CAR-T combination

**Fig. 5:**
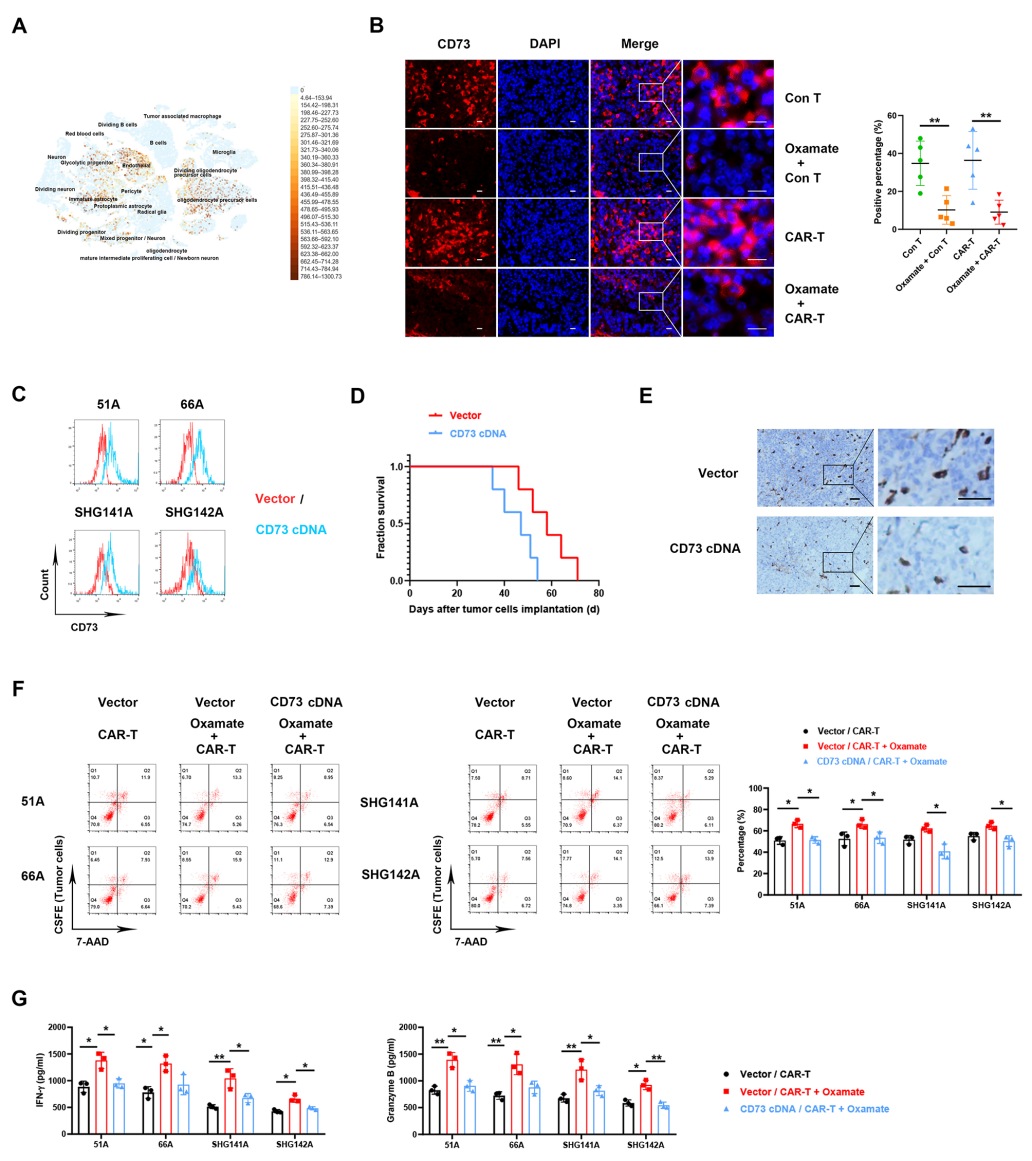
The effect of oxamate plus CAR-T was suppressed by CD73 overexpression in GSCs. (**A**) CD73 expression in GBM patients using single-cell RNA-seq. (**B**) CD73 expression by IF detection in the xenografts of nude mice. Scale bar represents 20 μm. (**C**) CD73 expression in GSCs was measured after vector or CD73 cDNA transfection. (**D**, **E**) 51 A GSCs transfected with vector or CD73 cDNA were orthotopically inoculated into nude mice to establish glioma model, then sodium oxamate and CAR-T were injected for antitumor therapy. N = 5. (**D**) Survival of nude mice was recorded. (**E**) Tumor infiltrating CD8 + T cells was stained 28 days after GSCs implantation. Scale bar represents 50 μm. (**F**) GSCs were prelabeled by CFSE, then co-cultured with CD8 + T cells at effector/target ratio of 5 : 1 for 16 h with or without 10 mM sodium oxamate. Dead cells were stained with 7-AAD and detected by flow cytometry. Double positive cells were dead tumor cells. (**G**) The concentration of IFN-γ and granzyme B were measured in co-cultured medium. *P < 0.05, **P < 0.01

The data of single-cell RNA-seq showed high expressions of CD73 in astrocyte, oligodendrocyte and endothelial cells. in GBM samples [19] (Fig. 5A). Our data also confirmed high expression of CD73 in GSCs grafts, and oxamate administration reduced CD73 expression (Fig. 5B). Furthermore, we inoculated nude mice with vector transfecting or CD73 overexpressing 51 A GSCs (Fig. 5C) to establish glioma models. After oxamate plus CAR-T treatment, a significant decrease of survival (Fig. 5D) was observed in nude mice implanted with CD73 overexpressing comparing with vector transfecting cells, and tumor infiltrating CD8 + T cells also reduced in xenografts (Fig. 5E). To explore whether oxamate promotes the efficacy of CAR-T due to CD73 downregulation in vitro, we co-cultured GSCs and CAR-T cells. Consistent with the results of animal model, oxamate enhanced the cytotoxic effect of CAR-T cells in vitro in 51 and 66 A GSCs (Fig. 5F), and a significant reduction on dead tumor cells was shown after oxamate plus CAR-T combination treatment in CD73 overexpressing compared with vector transfecting cells in all detected cells including 51 A, 66 A, SHG141A and SHG142A (Fig. 5F). Compared with vector group, the productions of IFN-γ and Granzyme B were also declined in co-cultured media in CD73 cDNA infection group (Fig. 5G). These data demonstrated that the effect of oxamate elevated CAR-T function via deceasing CD73 level.

### Lactate enhanced the interaction of CCR8 and its ligands on Treg cells and induced Th17/Treg imbalance

**Fig. 6:**
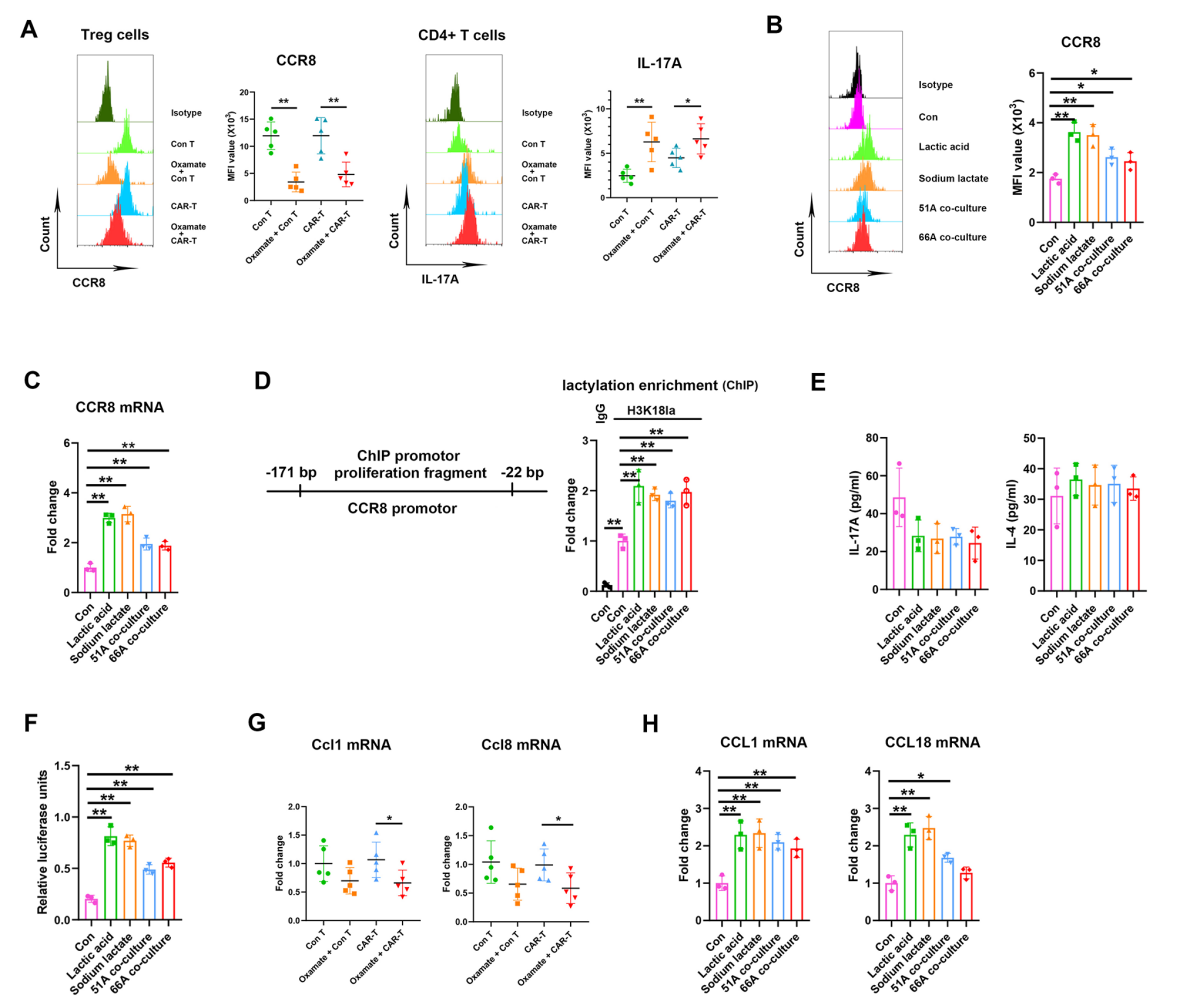
Lactate upregulated CCR8 level and led to Th17/Treg imbalance. (**A**) The levels of CCR8 in CD4 + CD25 + Treg cells and IL-17 A in CD4 + T cells within intracranial implanted tumor. CD4 + T cells isolated from human PBMCs were treated with lactic acid, sodium lactate, or co-cultured with 51 or 66 A GSCs, the expressions of CCR8 protein in CD4 + T cells (**B**), CCR8 mRNA expressions (**C**), H3K18la enrichment on CCR8 promotor (**D**), and the levels of IL-17 A and IL-4 in the supernatants (**E**) were measured. (**F**) CD4 + T cells were transfected with pGL3-CCR8 promotor plasmid for 24 h, then treated with PBS, lactic acid, sodium lactate or co-cultured with 51 or 66 A GSCs, the luciferase activities were measured by dual-luciferase reporter system. (**G**) The expressions of Ccl1 and Ccl8 mRNA in implanted tumor. (**H**) The expressions of CCL1 and CCL18 mRNA in THP-1 cells in vitro. *P < 0.05, **P < 0.01

The CCR8 is expressed principally on Treg cells within tumors [22]. Our analysis on immune cell subsets from separated tumor tissues showed high expressions of CCR8 on Treg cells in control T and CAR-T groups, oxamate treatment reduced CCR8 expressions, while elevated IL-17 A expressions in CD4 + T cells (Fig. 6A). CCR8 is not expressed in Treg in PBMCs [22], we further found increased expressions of CCR8 induced by lactate from cultured CD4 + T cells in vitro (Fig. 6B C), and elevated CCR8 promotor enrichments using anti-H3K18la antibodies (Fig. 6D). Oxamate blocked the effect of lactate on CCR8 expression and H3K18la on CCR8 promotor (Supplementary Fig S6). However, no differences were shown in the production of IL-17 A and IL-4 in the supernatants of cultured CD4 + T cells after lactate treatment (Fig. 6E). Moreover, luciferase reporter assay showed increased CCR8 promotor activity after lactate treatment or co-culture with GSCs (Fig. 6F). CCL1 and CCL18 produced by macrophages are known human CCR8 ligands with high affinity [23], and murine Ccl8 is a functional analog of human CCL18 [24]. Oxamate treatment decreased Ccl1 and Ccl8 expressions of tumor tissues from glioma implanted nude mice (Fig. 6G), consistently, CCL1 and CCL18 levels in cultured THP-1 cells in vitro were increased by lactate (Fig. 6H). These data suggested lactate in TME activated Treg through CCR8 pathway upregulation to enhance immunosuppression and disturbed Th17/Treg balance.

## Discussion

**Fig. 7:**
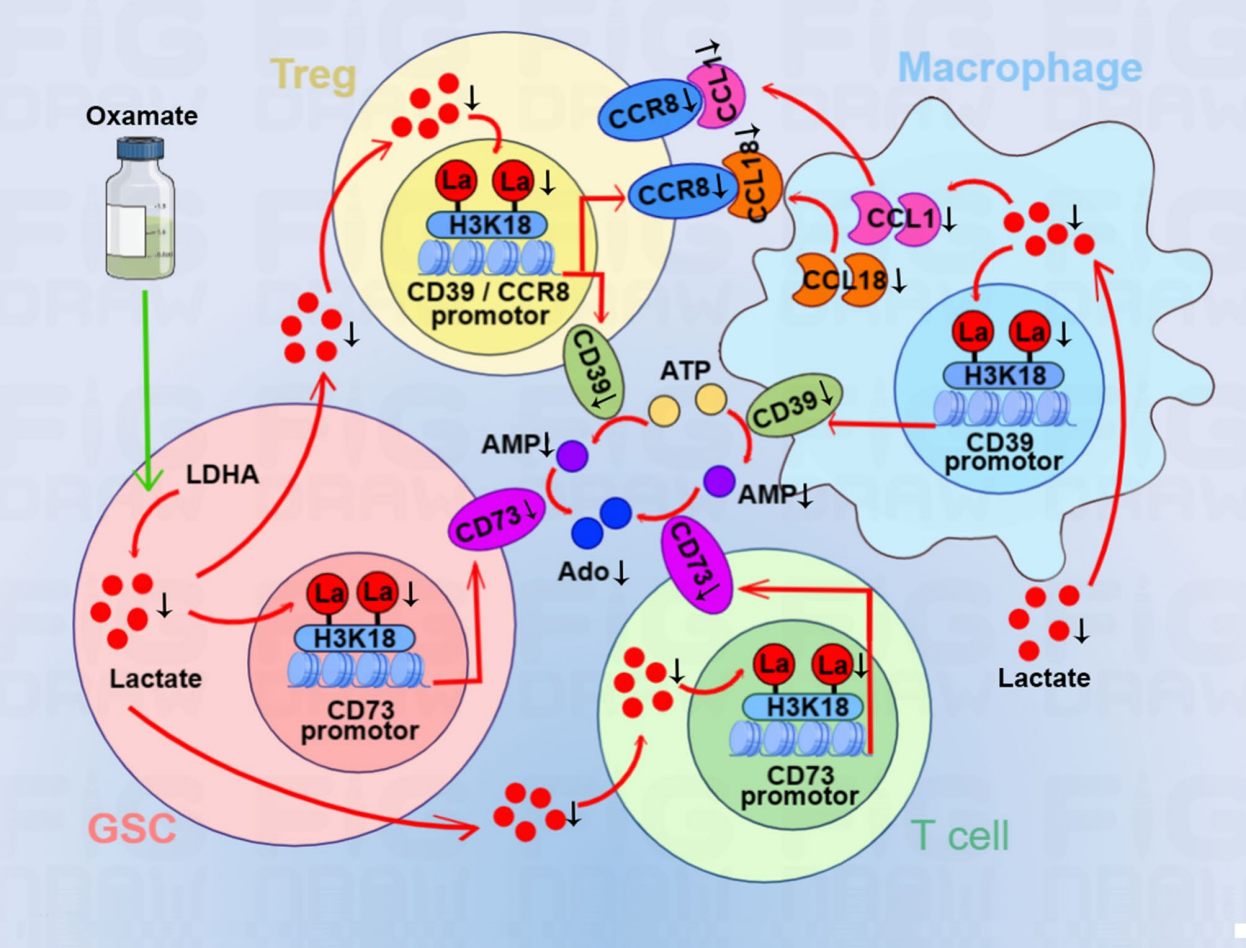
The mechanism of reduced intracellular lactate induced by oxamate leading to H3K18 lactylation on CD39, CD73 and CCR8 gene promotors. Oxamate inhibits lactate production and release into TME. Reduced lactate in GSCs and T cells suppresses histone H3K18 lactylation, leading to the decrease of CD73 gene promotor activity. Low level of H3K18 lactylation restrains the activity of CD39 gene promotor in Treg cells and macrophages. The conversion of ATP to AMP then to adenosine is inhibited by reduced levels of CD39 and CD73, respectively. Reduced CCR8 level due to low H3K18 lactylation blocks the binding to its ligands CCL1 and CCL18 secreted by macrophages. Oxamate changes the immunosuppressive microenvironment of GBM and improves the effect of CAR-T therapy

Utilizing lactate generation inhibitor modulates the activity of tumor and CAR-T cells simultaneously, which may be an effective strategy to enhance CAR-T function in GBM therapy. We found that oxamate altered the phenotypes of tumor-infiltrating CAR-T cells in GBM mouse model, including increased expressions of immunostimulating IFN-γ, cytotoxic perforin, and granzyme B. Furthermore, we confirmed that oxamate downregulated the levels of ectonucleotidases CD39 and CD73 for regulating immune microenvironment and CCR8 expressed in tumor-infiltrating Treg cells in CAR-T therapy. The in vitro experiments consistently demonstrated that lactate upregulated CD39 and CD73 levels and increased the expressions of CCR8 and its ligands CCL1 and CCL18 to disturb Treg/Th17 balance. In mechanism, lactate induced histone H3K18 lactylation to elevate the activities of CD39, CD73 and CCR8 genes promotors (Fig. 7). Therefore, combining oxamate and CAR-T therapy not only reprogrammed glucose metabolism of cancer cells but changed immunosuppressive TME by reducing adenosine production and tumor infiltrating Treg cells.

The accumulation of lactate and adenosine in TME are two major factors of immunosuppression, their concurrent downregulation has benefit for effective cancer immunotherapy. Extracellular ATP, an inflammatory signal, is converted to AMP by ectonucleotidases CD39 and then to adenosine by CD73 [25]. In this study, an LDHA inhibitor was used for decreasing lactate production to explore the effect of lactate concentration in TME on the regulation of CD39 and CD73. The data showed that LDHA inhibition decreased CD39 and CD73 expressions and elevated the antitumor function of CAR-T. We demonstrated that oxamate simultaneously decreased the concentration of lactate and adenosine by inhibiting CD39 and CD73 expressions, suggesting that oxamate is capable of reshaping an inflammatory microenvironment for CAR-T therapy.

Elevations of CAR-Treg cells were detected among nonresponders to CAR-T therapy, and these populations inhibited conventional CAR-T cells expansion and drove late relapses [26]. CCR8 upregulation was found in tumor-infiltrating Treg cells and correlated with poor prognosis in solid tumors, and targeting CCR8 for Treg depletion elicits antitumor immunity and synergizes with immunotherapy [27]. Our study demonstrated that oxamate reduced the number of tumor-infiltrating Treg cells through inhibiting CCR8 expression, lactate increased CCR8 expression in cultured CD4 + T cells in vitro. Consistent study confirmed that Treg cells withstood high concentration of lactate [28], the evidences indicated a positive correlation between lactate concentration in tumors and frequency of PD-1 + tumor-infiltrating Treg cells [29].

Energy production through glycolysis instead of the typical citric acid cycle and oxidative phosphorylation in cancer cells leads to the increase of lactate production [10]. High lactate level contributes to reprogramming metabolism of T cells to produce the exhausted phenotype and alter their function, which leads to the impairment of immune response [30]. Our finding linked lactate level in the TME to immune suppression and exhaustion and suggested that modulating TME to reduce lactate production was beneficial to improve glioma treatment. Elevated LDHA expression was found in cancer cells and tissues and related to worse prognosis in cancer patients [11]. LDHA inhibition reduced the growth and viability of cancer cells through hampering ATP production in glycolysis process, which was considered a suitable target for cancer therapy. In GBM mouse model, oxamate as an LDH inhibitor prevented the exhaustion and improved the efficacy of CAR-T cells during GBM treatment process, accompanying decreased expressions of PD-1 and Tim-3 in CAR-T cells. Previous study consistently demonstrated that lactate derived from glioma cells was abundant in the TME of glioma [31], and high concentration of lactate suppressed CD8 + T cell proliferation and cytokine production [32].

In addition to regulating immune cell function as a metabolite, lactate has recently been found as an epigenetic factor, and histone lactylation directly stimulates gene transcription [13] and promotes reparative macrophage transition [15]. Several studies have confirmed that lactate regulated histone H3K18 lactylation. Lactate accumulated potently induced METTL3 upregulation in tumor-infiltrating myeloid cells via H3K18 lactylation [33]. Pan-histone lactylation and specific H3K18 lactylation in Th17 cells resulted in the loss of IL-17 A production and shifted pro-inflammatory Th17 cells towards a regulatory T cell phenotype [21]. Additional study showed that histone lactylation at H4K12la site occurred in context microglia to drive pro-inflammatory microglial activation in Alzheimer’s disease [34] and neural excitation and social stress increased histone H1 lactylation [35]. Moreover, HMGB1 lactylation in macrophages [36] and MOESIN lactylation in Treg cells [37] demonstrated that lactation on protein lysine residues directly regulated protein expressions. Our research further found that histone lactylation upregulated CD39, CD73 and CCR8 expressions, which clarified a mechanism of immunosuppressive TME formation induced by the high concentration of lactate produced by tumor metabolism.

## Conclusions

In conclusion, oxamate inhibited lactate production and downregulated the activities of CD39, CD73 and CCR8 gene promotors through decreasing histone H3K18 lactylation. Oxamate altered immunosuppressive TME and promoted immune activation, suggesting that it may be a potential strategy to enhance CAR-T function in glioblastoma therapy.

### Electronic supplementary material

Below is the link to the electronic supplementary material.

Supplementary Material 1

Supplementary Material 2

## Data Availability

All cell lines, vectors and other stable reagents generated in this study are available from the corresponding author. The datasets used or analyzed are available on reasonable request.

## Abbreviations

**GBM**: Glioblastoma multiforme
**GSCs**: Glioma stem cells
**CAR-T**: Chimeric antigen receptor-T
**LDHA**: Lactate dehydrogenase A
**TME**: Tumor microenvironment
**Treg**: Regulatory T
**H3K18la**: Histone H3 lysine K18 lactylation
**CCR8**: Chemokine (C-C motif) receptor 8
**PBMCs**: Peripheral blood mononuclear cells
