# Supplementary material for: Oxamate enhances the efficacy of CAR-T therapy against glioblastoma via suppressing ectonucleotidases and CCR8 lactylation
Source: J Exp Clin Cancer Res. 2023 Sep 29;42:253. doi: 10.1186/s13046-023-02815-w (PMC10540361; doi:10.1186/s13046-023-02815-w)
Supplement: Supplementary file 1 — Supplementary Material 1 [file 13046_2023_2815_MOESM1_ESM.docx]

**Oxamate enhances the efficacy of CAR-T therapy against glioblastoma via suppressing ectonucleotidases and CCR8 lactylation**

Ting Sun1*****, Bin Liu2, Yanyan Li1, Jie Wu1, Yufei Cao1, Shuangyu Yang3, Huiling Tan3, Lize Cai1, Shiqi zhang1, Xinyue Qi1, Dingjia Yu1, Wei Yang3*****

1. Neurosurgery and Brain and Nerve Research Laboratory, The First Affiliated Hospital of Soochow University, Suzhou, Jiangsu, China.

2. Department of Neurosurgery, Qinghai Provincial People’s Hospital, Xining, Qinghai, China.

3. State Key Laboratory of Radiation Medicine and Protection, School of Radiation Medicine and Protection and Collaborative Innovation Center of Radiation Medicine of Jiangsu Higher Education Institutions, Soochow University, Suzhou, Jiangsu, China.

Ting Sun, Bin Liu and Yanyan Li contributed equally to the work.

Corresponding Authors: Ting Sun, E-mail: sunting1979st@aliyun.com; Wei Yang, E-mail: detachedy@aliyun.com

**Supplementary Figures**





**Fig S1.** **Identification of macrophage, CD3+ and CD4+ T lymphocytes.** The purity of THP-1 differentiated macrophages using human CD163 antibody (A), CD3+ T cells (B) and CD4+ T cells (C) isolated from PBMCs.


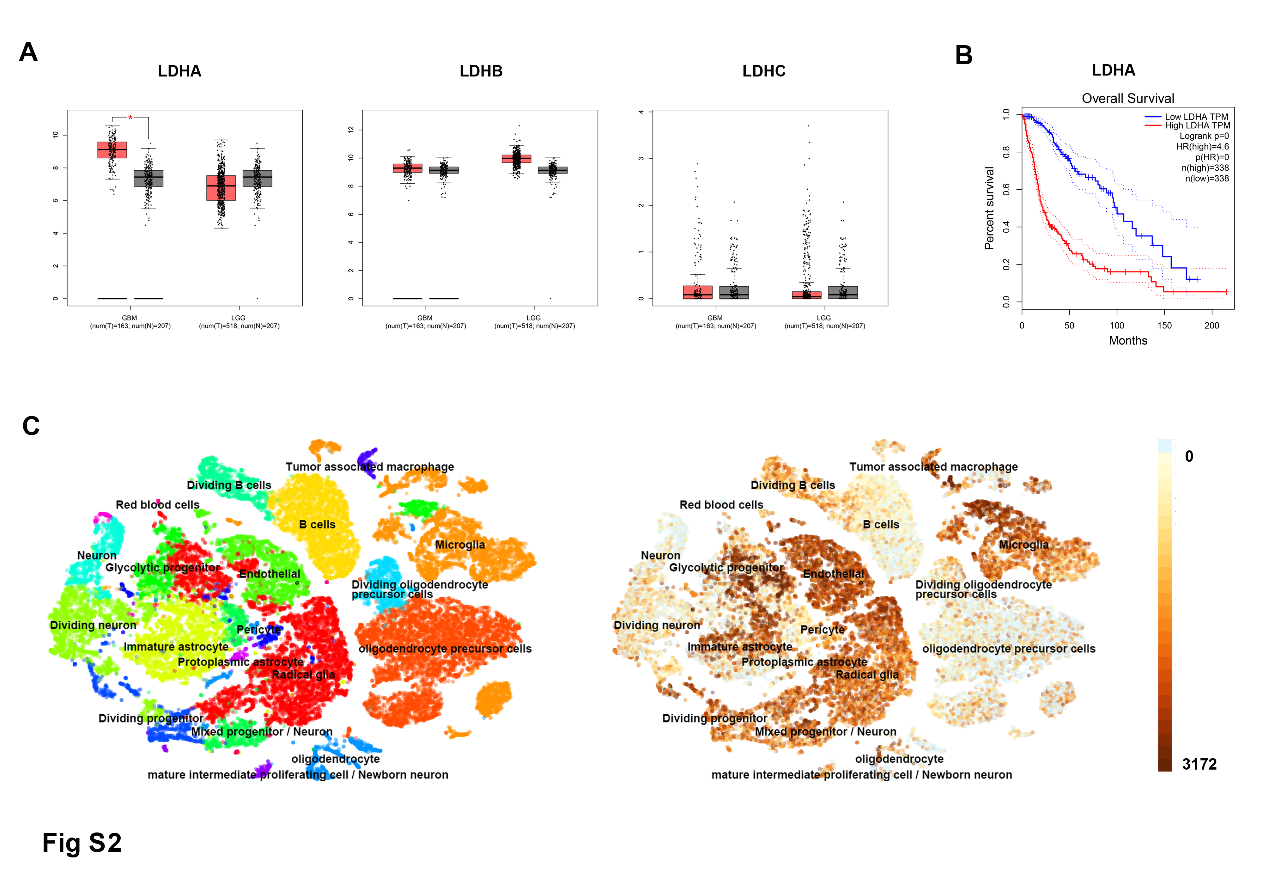


**Fig S2. The analysis of LDHA expressions in glioma patients in public databases.**

(A) The expression levels of LDHs including LDHA, LDHB and LDHC in GBM and LGG patients in TCGA dataset. (B) Overall survival plots of GBM and LGG patients grouped into LDHA high-expression and low-expression group based on median expression in TCGA dataset. (C) Transcriptomic expression of LDHA using single-cell sequencing. *P* < 0.05.


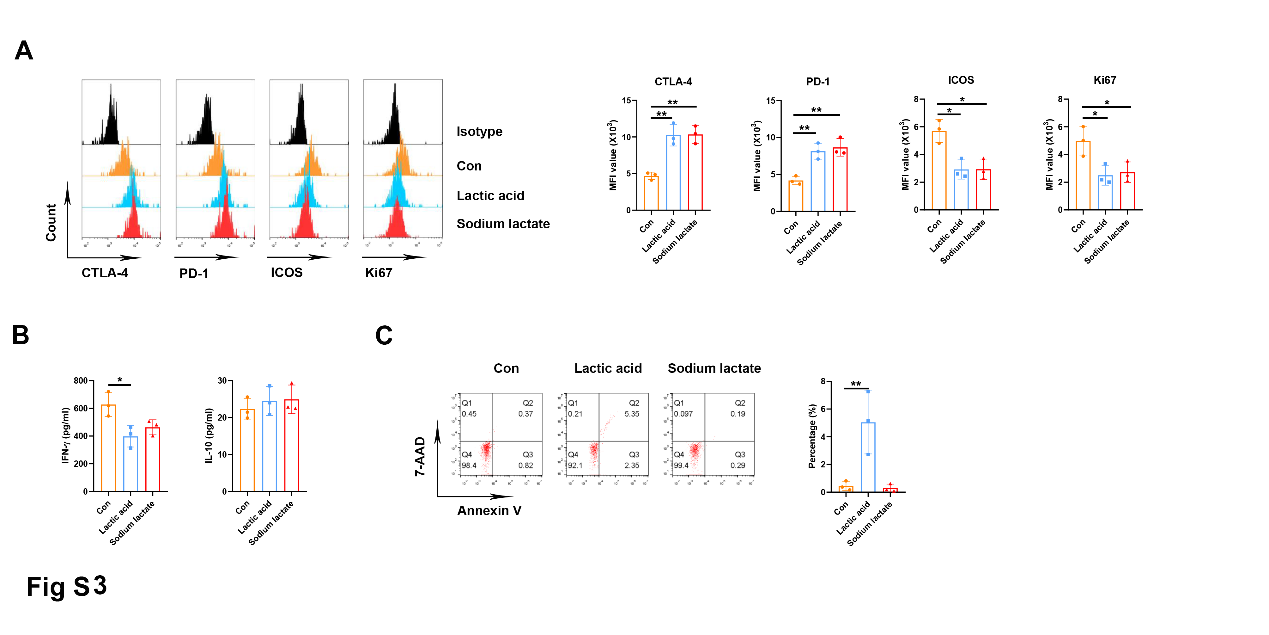


**Fig S3. Lactate inhibits the pro-inflammatory function of T lymphocytes.**

T lymphocytes were treated with PBS, 10 mM lactic acid or sodium lactate for 24 h. (A) The expressions of CTLA-4, PD-1, ICOS and Ki-67 were detected using flow cytometry. (B) The concentration of IFN-γ and IL-10 in supernatants were measured using ELISA. (C) Cells were stained with Annexin V and 7-AAD, double positive cells were calculated as death cells. **P* < 0.05, ***P* < 0.01.


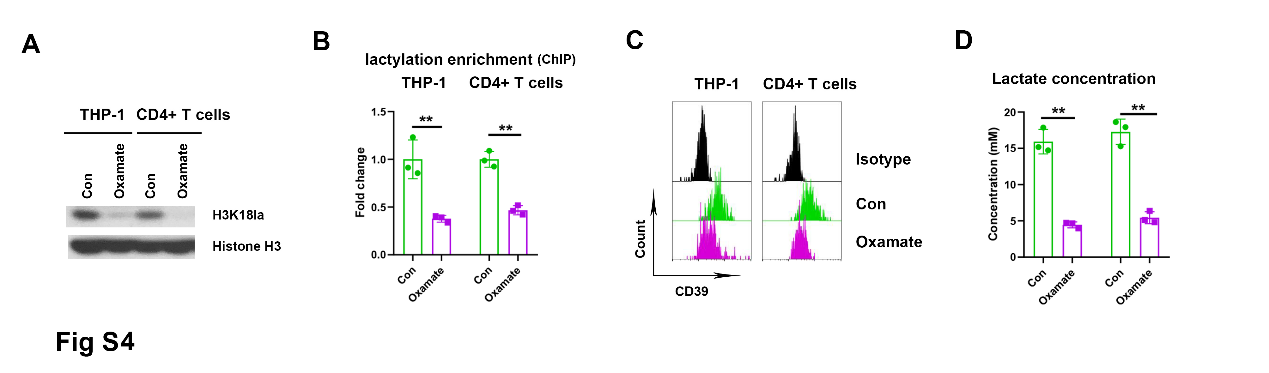


**Fig S4. Oxamate decreased CD39 expression by reducing H3K18 lactylation.** 51A GSCs were co-cultured with THP-1 or CD4+ T cells, 20 mM sodium oxamate or PBS was added into medium for 24 h. Oxamate decreased H3K18la level using western blot (A), H3K18la enrichments on CD39 promoter using ChIP assay (B), CD39 expression using flow cytometry (C) and lactate concentration (D) were measured in THP-1 and CD4+ T cells . **P* < 0.05, ***P* < 0.01.


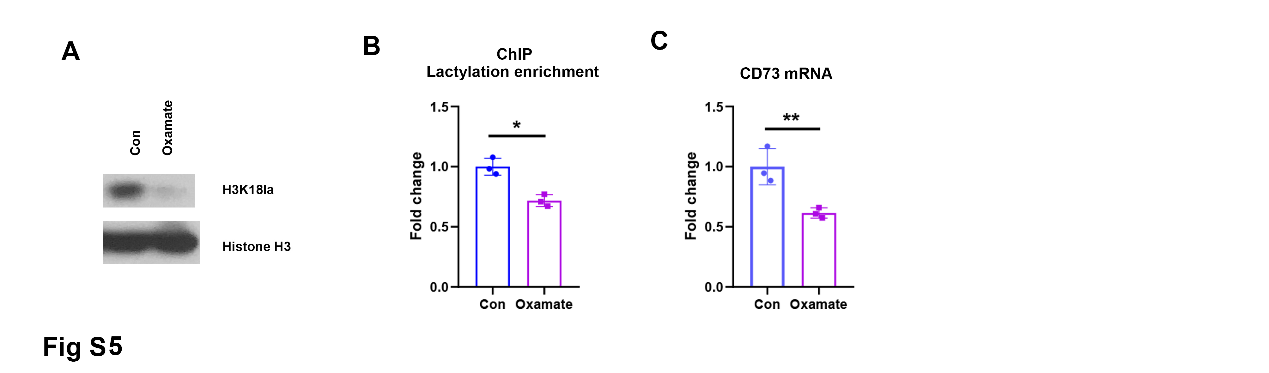


**Fig S5. Oxamate decreased CD73 expression by reducing H3K18 lactylation.** 51A GSCs were co-cultured with isolated T cells, sodium oxamate or PBS was added into medium for 24 h. H3K18la level (A), H3K18la enrichments on CD73 promoter (B) and CD73 mRNA expression (C) were measured in T cells . **P* < 0.05, ***P* < 0.01.


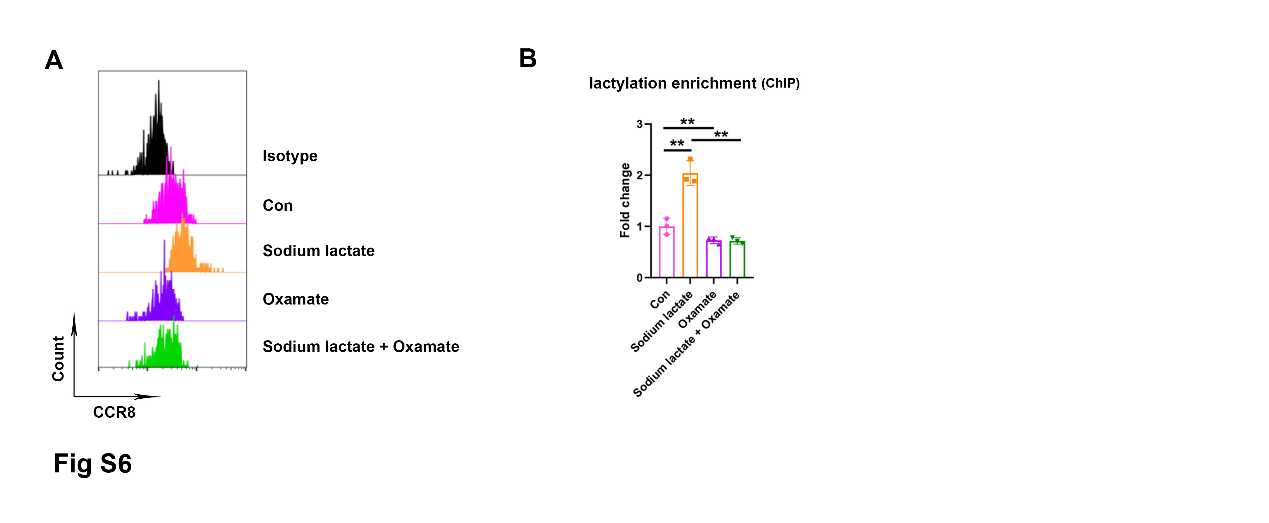


**Fig S6. Oxamate blocked H3K18 lactylation on CCR8 promotor induced by lactate in CD4+ T cells.** Isolated CD4+ T cells were treated with sodium lactate, sodium oxamate or combination. the expressions of CCR8 protein using flow cytometry (A) and H3K18la enrichment on CCR8 promotor using ChIP assay (B) were detected. **P* < 0.05, ***P* < 0.01.
