# Supplementary material for: Oxamate enhances the efficacy of CAR-T therapy against glioblastoma via suppressing ectonucleotidases and CCR8 lactylation
Source: J Exp Clin Cancer Res. 2023 Sep 29;42:253. doi: 10.1186/s13046-023-02815-w (PMC10540361; doi:10.1186/s13046-023-02815-w)
Supplement: Supplementary file 2 — Supplementary Material 2 [file 13046_2023_2815_MOESM2_ESM.docx]

**Supplementary Tables**

**Supplementary Table 1. Primers Sequences Table**

| Name | Species | Sequences |
| --- | --- | --- |
| CD39 (ChIP) | human | Forward: 5’-TTGGCTCATGAGAAGGCTGA-3′ |
|  |  | Reverse: 5’-AAGGGCTTCTTCGTCACTGA-3′ |
| CD73 (ChIP) | human | Forward: 5’-GGGTGATGGGTATGTGGGAA-3’ |
|  |  | Reverse: 5’-CCAAGAAAGGCCATGCAGTT-3’ |
| CCR8 (ChIP) | human | Forward: 5’-ACAGGTCAGTGGCTCAATCA-3’ |
|  |  | Reverse: 5’-AGCAGTGTGACAACGGACTA-3’ |
| CD39 (mRNA) | human | Forward: 5’-AGGTGCCTATGGCTGGATTAC-3’ |
|  |  | Reverse: 5’-CCAAAGCTCCAAAGGTTTCCT-3’ |
| CD73 (mRNA) | human | Forward: 5’-CCAGTACCAGGGCACTATCTG-3’ |
|  |  | Reverse: 5’-TGGCTCGATCAGTCCTTCCA-3’ |
| CCR8 (mRNA) | human | Forward: 5’-GTGTGACAACAGTGACCGACT-3’ |
|  |  | Reverse: 5’-CTTCTTGCAGACCACAAGGAC-3’ |
| CCL18 | human | Forward: 5’-CCTGTGCACAAGTTGGTACC-3’ |
|  |  | Reverse: 5’-ATTGGGGTCAGCACAGATCT-3’ |
| CCL1 | human | Forward: 5’-CTCATTTGCGGAGCAAGAGAT-3’ |
|  |  | Reverse: 5’-GCCTCTGAACCCATCCAACTG-3’ |
| GAPDH | human | Forward: 5’-GGAGCGAGATCCCTCCAAAAT-3’ |
|  |  | Reverse: 5’-GGCTGTTGTCATACTTCTCATGG-3’ |
| Ccl1 | Mouse | Forward: 5′-GGCTGCCGTGTGGATACAG-3′ |
|  |  | Reverse: 5′-AGGTGATTTTGAACCCACGTTT-3′ |
| Ccl8 | Mouse | Forward: 5′-TCTACGCAGTGCTTCTTTGCC-3’ |
|  |  | Reverse: 5′-AAGGGGGATCTTCAGCTTTAGTA-3’ |
| Gapdh | Mouse | Forward: 5’-AAATGGTGAAGGTCGGTGTG-3’ |
|  |  | Reverse: 5’-TGAAGGGGTCGTTGATGG-3’ |

Supplementary Table 2. Product Table

| BD Horizon™ BV605 Mouse Anti-Human CD45 | BD Biosciences | 564047 |
| --- | --- | --- |
| BD Horizon™ BV711 Mouse Anti-Human CD3 | BD Biosciences | 563725 |
| BD Pharmingen™ PerCP-Cy™5.5 Mouse Anti-Human CD4 | BD Biosciences | 552838 |
| BD Horizon™ BV510 Mouse Anti-Human CD8 | BD Biosciences | 563256 |
| CD25 Monoclonal Antibody (BC96), eFluor™ 450 | eBioscience | 48-0259-42 |
| CD39 Monoclonal Antibody (eBioA1 (A1)), PE | eBioscience | 12-0399-42 |
| CD73 Monoclonal Antibody (AD2), FITC | eBioscience | 11-0739-42 |
| BD OptiBuild™ BV786 Mouse Anti-Human CCR8 (CD198) | BD Biosciences | 747574 |
| Perforin Monoclonal Antibody (dG9 (delta G9)), APC | eBioscience | 17-9994-42 |
| Granzyme B Monoclonal Antibody (GB11), PE | eBioscience | 12-8899-41 |
| BD Horizon™ BV650 Mouse Anti-Human IFN-γ | BD Biosciences | 563416 |
| BD Pharmingen™ FITC Rat Anti-CD11b | BD Biosciences | 561688 |
| BD Horizon™ BV605 Rat Anti-Mouse CD45 | BD Biosciences | 563053 |
| BD OptiBuild™ BV650 Rat Anti-Mouse F4/80 | BD Biosciences | 743282 |
| BD Pharmingen™ APC Rat Anti-Mouse Ly-6C | BD Biosciences | 560595 |
| BD OptiBuild™ BV711 Mouse Anti-Human CCR8 (CD198) | BD Biosciences | 747575 |
| CD39 Monoclonal Antibody (eBioA1 (A1)), PerCP-eFluor™ 710 | eBioscience | 46-0399-42 |
| CD39 Monoclonal Antibody (24DMS1), PE-Cyanine7 | eBioscience | 25-0391-82 |
| CD73 Monoclonal Antibody (AD2), PE | eBioscience | 12-0739-42 |
| CD152 (CTLA-4) Monoclonal Antibody (14D3), PE | eBioscience | 12-1529-42 |
| Anti-CD4 antibody [EPR6855] | Abcam | ab133616 |
| Anti-CD8 alpha antibody [C8/144B] | Abcam | ab17147 |
| Lactyl-Histone H3 (Lys18) Rabbit pAb | Ptm Biolabs | PTM-1406 |
| Histone H3 (D1H2) XP® Rabbit mAb | Cell Signalling  Technology | 4499 |
| CD3 Monoclonal Antibody (OKT3), Functional Grade, | eBioscience | 16-0037-81 |
| CD28 Monoclonal Antibody (CD28.2), Functional Grade | eBioscience | 16-0289-81 |
| Tumor Dissociation Kit | Miltenyi Biotec | 130-096-730 |
| ACK Lysing Buffer | Gibco | A1049201 |
| BD Leukocyte Activation Cocktail, with BD GolgiPlug™ | BD Biosciences | 550583 |
| BD Cytofix/Cytoperm™ Plus Fixation/Permeabilization Kit (with BD GolgiPlug^TM^) | BD Biosciences | 555028 |
| LIVE/DEAD™ Fixable Blue Dead Cell Stain Kit, for UV excitation | Invitrogen | L34962 |
| CellTrace™ CFSE Cell Proliferation Kit | Invitrogen | C34554 |
| FITC Annexin V Apoptosis Detection Kit with PI | Biolegend | 640914 |
| Human IFN-γ Elisa Kit | Biolegend | 430107 |
| Human Granzyme B ELISA development kit | MabTech | 3485-1A-6 |
| SimpleChIP® Enzymatic Chromatin IP Kit | Cell signaling | 9002 |
| Lactate Colorimetric Assay Kit | Biovision | K627-100 |
| EasySep™ Human CD8+ T Cell Isolation Kit | STEMCELL Technologies | 17953 |
| EasySep™ Human CD4+ T Cell Isolation Kit | STEMCELL Technologies | 17952 |
| EasySep™ Human T Cell Isolation Kit | STEMCELL Technologies | 17951 |
| Dual-Glo Luciferase Assay System | Promega | E2920 |
| BCA Protein Assay Kit | Beyotime Biot | P0010 |
| Pierce™ ECL Western Blotting Substrate | Thermo Scientific | 32106 |
| RevertAid First Strand cDNA Synthesis Kit | Thermo Scientific | K1622 |
| PowerUp™ SYBR™ Green Master Mix | Applied Biosystems | A25742 |
| TRIzol™ Reagent | Invitrogen | 15596026 |
| Sodium oxamate | Sigma-Aldrich | 02751 |
| L-lactic acid | Sigma-Aldrich | L1750 |
| L-Sodium lactate | Sigma-Aldrich | L7022 |
| CHC, Monocarboxylic acid transport inhibitor | Abcam | ab146008 |
| D-Luciferin | Sigma-Aldrich | L9504 |
| 6.5 mm Transwell with 0.4 um pore polycarbonate Membrane Insert, Sterile | Corning | 3413 |
